# Ageism Linked to Culture, Not Demographics: Evidence From an 8-Billion-Word Corpus Across 20 Countries

**DOI:** 10.1093/geronb/gbaa181

**Published:** 2020-10-25

**Authors:** Reuben Ng, Jeremy W Lim-Soh

**Affiliations:** 1 Lee Kuan Yew School of Public Policy, National University of Singapore; 2 Lloyds Register Foundation Institute for the Public Understanding of Risk, National University of Singapore

**Keywords:** Age stereotypes, Long-term orientation, Masculinity, Psychomics, Quantitative social science

## Abstract

**Objectives:**

Ageism has increased over 200 years and costs the U.S. health care system $63 billion a year. While scholars agree on the consequences of ageism, there are disagreements on whether it is related to the demographics of aging, or society’s cultural values. We test both hypotheses across 20 countries.

**Method:**

To circumvent the sampling limitations of survey studies, we used an 8-billion-word corpus, identified 3 synonyms with the highest prevalence—aged, elderly, old people—and compiled the top 300 words (collocates) that were used most frequently with these synonyms for each of the 20 countries. The resulting 6,000 collocates were rated on an ageism scale by 2 raters to create an ageism score per country. Cultural dimension scores—Power Distance, Individualism, Masculinity, Uncertainty Avoidance, and Long-term Orientation—were taken from Hofstede, and demographics—size and speed of population aging—came from the World Development Indicators.

**Results:**

Of the 20 countries, UK topped the ageism table, while Sri Lanka had the lowest ageism score. Multiple regression models showed that higher levels of masculinity and long-term orientation are associated with ageism, controlling for other cultural dimensions, demographics (size and speed of aging), and economics (GDP-per-capita).

**Discussion:**

Our findings blunt the deterministic nature of ageism at the societal level. Demographics is only one side of the ageism coin, and the cultural side is equally, if not more important. This study lays the groundwork to tackle societal ageism—one of our generation’s most pernicious threats.

The negative impact of ageism on health and well-being is well established, but there are disagreements on the factors that promote ageism. There are two competing hypotheses. First, the demographic argument linked ageism to the size of the aging population, as posited by Löckenhoff et al. (2009), as well as the speed of aging (North & Fiske, 2015). Second, the cultural argument linked ageism to one’s “social environment” (Hofstede, 2001, p. 6), albeit with mixed results across life domains and age groups (North & Fiske, 2015; Voss et al., 2018). Though many studies have advanced the competing hypotheses in a mutually exclusive way, few have tested both demographic and cultural hypotheses in the same study. A systematic review of ageism across 199 papers found that only eight studies considered institutional or cultural determinants (Marques et al., 2020).

We aim to test the competing hypotheses across 20 countries through a computational linguistics approach that measures societal ageism in a cross-cultural corpus of 8 billion words. According to Cultivation Theory (Gerbner et al., 1994), the large representation of online media within the corpus reflects societal perceptions of respective countries and provide an extraordinary platform to study ageism. An example of such an approach is Ng et al.’s (2015) study of how age stereotypes have changed over time in a historical corpus of 400 million words that spans 200 years. They found that age stereotypes became more negative over time, and switched from positive to negative after 1880.

This study is significant in three ways: (a) Conceptually, the cultural antecedents of ageism cannot be studied independently of the demographic and economic realities that differentiate countries. Our study provides a nuanced view of the cultural predictors of ageism, after adjusting for demographic and economic covariates. (b) The innovative use of our country-level corpus to measure ageism circumvents the sampling limitations of survey studies (Hofstede, 2001, p. 4). (c) Practically, understanding the cultural underpinnings of ageism lays the groundwork for designing interventions to reduce it, as studies shows the malleability of cultural frames (e.g., Dweck et al., 1995).

## Cultural Dimensions

Hofstede (1984, 2001) conceptualized one of the most comprehensive and widely used dimensions of national culture through surveys of IBM employees in 72 countries and validated his work with subsequent surveys. Though Hofstede’s dimensions have been criticized (as much as they have been cited; Minkov, 2018), they remain useful for this study. First, the multiple dimensions provide a comprehensive coverage of cultural differences, as compared to any single dimension. Second, they are widely recognized and understood, demonstrating this study’s comparability and contribution to prior literature. Third, the Hofstede dimensions are intended as a national-level (between-system) measurement of culture as opposed to other individual-level (within-system) cultural variables (Hofstede, 2001; Zhang et al., 2016). Since our platform measures age stereotypes at the national level, we found Hofstede’s dimensions to be compatible as they conceptualize culture at the national level.

The framework started with four dimensions—Power Distance, Individualism, Masculinity, and Uncertainty Avoidance. Subsequently, Long-term Orientation was added to accommodate the new findings from the Chinese Values Survey of Asian cultures (Hofstede, 2001). *Power Distance* is “the extent to which the less powerful members of organizations and institutions accept and expect that power is distributed unequally. The basic problem involved is the degree of human inequality that underlies the functioning of each particular society.” *Individualism* is “the degree to which individuals are supposed to look after themselves or remain integrated into groups, usually around the family. Positioning itself between these poles is a very basic problem all societies face.” *Masculinity* is “the distribution of emotional roles between the genders, which is another fundamental problem for any society to which a range of solutions are found; it opposes ‘tough’ masculine to ‘tender’ feminine societies.” *Uncertainty Avoidance* is “the extent to which a culture programs its members to feel either uncomfortable or comfortable in unstructured situations. Unstructured situations are novel, unknown, surprising, different from usual. The basic problem involved is the degree to which a society tries to control the uncontrollable.” *Long-term Orientation* is “the extent to which a culture programs its members to accept delayed gratification of their material, social, and emotional needs.” (Hofstede, 2001, p. xix).

## Masculinity, Long-term Orientation, Individualism, and Ageism

There are no known studies on the associations between Masculinity, Long-term Orientation, and age stereotypes at the national level, though current studies point to potential links. Existing qualitative studies found a link between Masculinity and ageism in the IT industry, which is dominated by men, with a sizable proportion who are reaching older age. Interviews revealed that older IT workers are perceived negatively due to their age and decreased Masculinity since they are perceived to be trailing their younger male colleagues (Comeau & Kemp, 2007). Another qualitative study in older men with chronic conditions also found that illness brought about a perception of deceased Masculinity and increased ageism (Separavich & Oliveira, 2020). In contrast, a survey study of undergraduates found that stereotypes of older men are positive, and increasingly so with perceptions of Masculinity (Thompson, 2006). Building on these qualitative and survey studies, we hypothesize that countries with higher reported Masculinity will be associated with more negative age stereotypes (Hypothesis 1).

Increased Long-term Orientation has been linked to more proactive security actions (Aurigemma & Mattson, 2019), innovation (Bukowski & Rudnicki, 2019), and resilience (Sulphey, 2020). These studies show that Long-term Orientation involves the delay of current gratification for a future benefit such as increased security and innovation. However, this delayed approach is unrealistic for aging societies that have to deal proactively with the needs of the elderly population *immediately* or risk spending more for acute care later. Against this background, we hypothesize that higher Long-term Orientation will be associated with negative age stereotypes (Hypothesis 2).

With regard to Collectivism and age stereotypes, North and Fiske (2015) found that collectivism is associated with more negative age stereotypes, as it could spur resentment toward older adults for demanding support and enjoying benefits without contributing to society. In line with these findings, we hypothesize that increased Collectivism will be associated with more negative age stereotypes (Hypothesis 3).

## Demographics and Ageism

North and Fiske (2012, p. 987) suggested that one cause of ageism could be the “resource threat” posed by the elderly who form an increasingly large proportion of the population, and may be seen to contribute to the fiscal burden (Ng, Lim, et al., 2020; Tepe & Vanhuysse, 2009; Walker, 1990). Löckenhoff et al. (2009), in survey of college students across 26 countries, found that the size of the above-65 population was related to ageism, while North and Fiske (2015) in a meta-analysis of 37 East-West comparisons found that the speed of aging—rate of growth in the population above 65 years—was related to ageism. The implications of the demographic thesis are very different from those of the cultural thesis (Hypotheses 1, 2, and 3). As population aging is an inevitable demographic reality in most developed countries, a validation of the demographic thesis would suggest that we should be resigned to the growth of ageism at the societal level. On the other hand, culture has proven to be surprisingly malleable in priming studies (Dweck et al., 1995), including experiments that increased the physical performance of older adults (Levy et al., 2014), and decreased interpersonal ageism between undergraduate students and older adults (Lytle et al., 2020). We sought to test both theses in the same model.

## Method

### Dataset

The News on the Web Corpus (Davies, 2017) is the largest cross-cultural corpus that consists of web-based newspapers and magazines from 7,000 websites across 20 countries with over 28 million articles. This dataset—created with funding from the National Science Foundation (NSF) and the National Endowment for the Humanities (NEH) to study contemporary language usage in countries where English is widely used—is appropriate as Cultivation Theory (Gerbner et al., 1994) suggests that the large representation of online media reflects the societal perceptions of respective countries and provides an extraordinary platform to study societal ageism. For this study, we used a full year’s dataset (2017) with 1.75 billion words. The geographical makeup of the corpus spans six regions: North America (America and Canada); the British Isles (Ireland and United Kingdom); Oceania (Australia and New Zealand); Asia (Bangladesh, Hong Kong, India, Malaysia, Pakistan, Philippines, Singapore, and Sri Lanka); Africa (Ghana, Kenya, Nigeria, South Africa, and Tanzania); the Caribbean (Jamaica).

### Measurement of Ageism

We used three synonyms that were consistent with Ng et al., (2015) study, and evidenced the highest normalized frequencies in the dataset: “aged” (41.5 per million), “elderly” (22.2 per million), and “old people” (1.3 per million). For each synonym, we compiled the top 300 words that co-occurred most frequently, known as collocates, for each of the 20 countries with the following inclusion criteria: (a) Lexical Proximity: collocate present within four words prior or after the respective synonym; (b) Relevant context: collocate referred to specifically to an old person (checked by two raters); and (c) Mutual Information Score of 3 and above: collocate had a stronger association with the respective synonym than other words in the corpus for that country indicating semantic bonding (Church & Hanks, 1990). This is an innovative application of concordance analysis, used in computational linguistics to study language shifts and to identify stereotypes in other studies (Ng et al., 2015). The rigorous process generated 6,000 (300*20) collocates. Thereafter, each collocate that met the study criteria was rated on a scale from 1 (very positive) to 5 (very negative), a method found to be valid and reliable to measure age-stereotype associated words (Levy & Langer, 1994). For example, ‘frail’ will be rated as very negative while ‘wise’ would be rated as very positive. The inter-rater reliability using Cronbach’s alpha was .991 (95% CI: 0.987, 0.994) for the scoring method. Age stereotypes (ageism scores) for each country were created by calculating the mean of all 300 collocate scores for that country.

### Hofstede’s Five Cultural Dimensions

Calculations of the country dimension scores are found in Hofstede (2001) and Hofstede and Minkov (2013) that were based on the original IBM surveys, and subsequent studies (e.g., Hofstede Insights, 2020). The country score for each dimension is calculated as follows. First, the individual survey responses to each question are summed up at the national level. For a question requiring a 5-point Likert scale, this is done by assigning 1–5 points for each answer and then taking the national mean of the answers. For a question requiring a Yes/No or multiple-choice answer, this is done by taking the national percentage who gave a specific answer or set of answers, such “Option A OR Option C.” Second, these national-level question scores are combined according to a weighted formula to yield a country dimension score that is based on three to eight survey questions. The weights are used to balance the importance of each survey question as well as generate final scores that range from 0 to 100. For the present analysis, the scores were divided by 100 to match our other variables, such that they range from 0 to 1.

### Demographic Variable: Aging

To test the demographic hypothesis that ageism is related to the size of the elderly population, we included the size of the above-65 population as a proportion of the total population, following previous studies (e.g., Löckenhoff et al., 2009). Rather than the percentage above 65 years, North and Fiske (2015) argued that ageism is associated with a country’s speed of aging as a faster decline in the old age support ratio—or the average number of working adults who are supporting a retiree—will generate more intense fiscal pressures that pay out pensions at the expense of young taxpayers. We found this argument to be valid and included both the proportion above 65 years and the speed of aging for the respective country. The latter is calculated by subtracting the 2007 statistic for the size of the above-65 population from the 2017 statistic in the World Development Indicators (World Bank, 2020), which is a cross-country panel dataset that covers developmental statistics.

### Controls: GDP per capita

Prior studies (Löckenhoff et al., 2009; North & Fiske, 2015) controlled for GDP per capita to test the possible influence of level of development or modernization on ageism. Similarly, we used a measure of logged GDP per capita from the World Development Indicators (World Bank, 2020) as covariates.

### Analytic Strategy

To test the competing hypotheses of culture and demographics, we ran a series of OLS regressions with the cultural and demographic variables as predictors and age stereotypes as the outcome. We ran four models to establish robustness. Model 1 included the percentage above 65 years, and controlled for GDP per capita. Model 2 used the speed of aging in the past decade as the demographic predictor, and controlled for GDP per capita. Model 3 included only the cultural variables, controlling for GDP per capita, and Model 4 is the full model that included all demographic and cultural variables, controlling for GDP per capita. As Long-term Orientation scores were unavailable for Jamaica and Kenya, complete-case analysis was applied for Models 3 and 4. We tested for heteroskedasticity using visual confirmation of the residual-versus-fitted plot of the main model as well as White’s and Breusch–Pagan / Cook–Weisberg tests. All statistical analyses were conducted in SPSS 25.

## Results

### Descriptive Statistics: Ageism and Cultural Dimensions

The descriptive statistics for all variables are as follows. The dependent variable, age stereotypes, had a theoretical range of 1 (most positive) to 5 (most negative). However, the values in the sample ranged from 2.79 to 3.37. Of the 20 countries/territories, 17 evidenced negative age stereotypes, with the UK as the most negative. Age stereotypes are positive in three countries, Ghana, Tanzania, and Sri Lanka, with the latter being the most positive. This corroborates the literature on the ubiquity of ageism around the world (Ayalon & Tesch-Romer, 2018).

**Figure 1.**
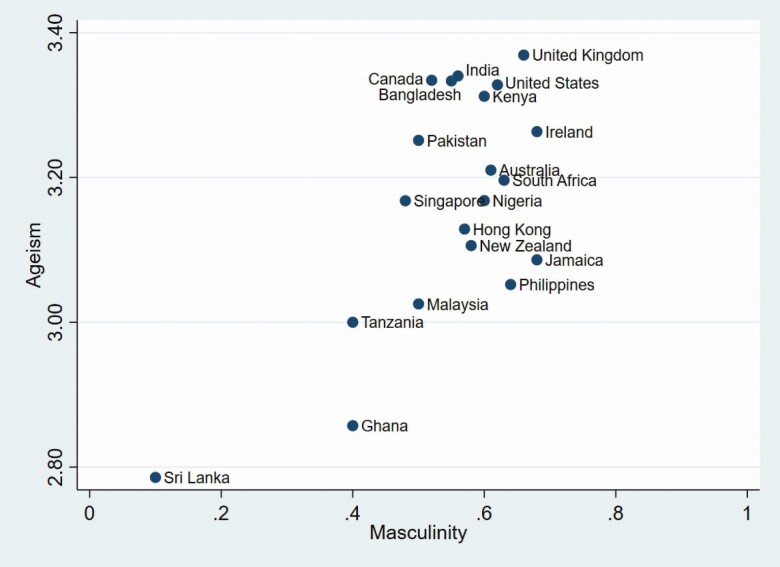
Scatterplot of ageism and masculinity across 20 countries.

**Figure 2.**
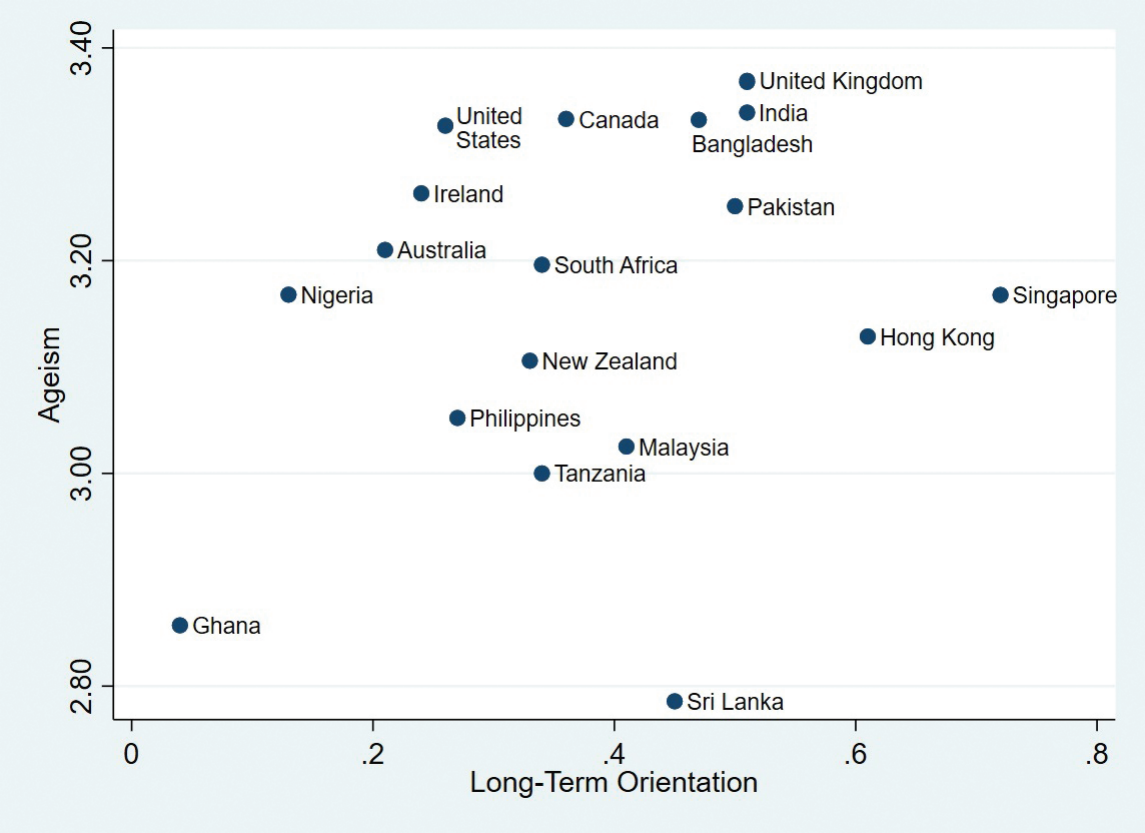
Scatterplot of ageism and long-term orientation across 20 countries.

The five cultural dimensions had a theoretical range from 0 to 1, with means ranging from 0.44 to 0.61, and standard deviations ranging from 0.13 to 0.28. The territory with the lowest Power Distance was New Zealand, while the highest was Malaysia. The territory with the lowest Collectivism was the United States, while the highest was Pakistan. The territory with the lowest Masculinity was Sri Lanka, while the highest was a tie between Jamaica and Ireland. The territory with the lowest Uncertainty Avoidance was Singapore, while the highest was Pakistan. Singapore evidenced the highest scores in Long-term Orientation while Ghana scored the lowest. Scatter plots for the relationships between age stereotypes, Masculinity, and Long-term Orientation, that relate to our hypotheses, are presented in Figures 1 and 2.

The mean population above 65 years was 9%, which is approximately the global average. The territory with the largest population above 65 was the United Kingdom (18%), and the one with the smallest was Kenya (2%). Meanwhile, the mean change in population above 65 years over 10 years was 2%, with the fastest aging in Hong Kong (4%), and no aging trend in Nigeria (0%). This shows that our sample covers a good range of values for the demographic hypotheses.

### Multivariable Regression

We tested the hypotheses progressively across four models. Models 1 and 2 tested the demographic hypothesis that aging societies are associated with increased ageism. Neither “percentage of elders above 65 years” (Model 1) nor speed of aging measured by “change of population above 65 years” reached significance, controlling for GDP per capita. Therefore, we did not find evidence in support of Hypotheses 4 and 5.

Through Model 4, we found support for Hypotheses 1 and 2. Masculinity was significantly associated with ageism (β = .85, *p* < .01), and Long-term Orientation was associated with ageism (β = .65, *p* < .05) controlling for other cultural dimensions (Power Distance, Collectivism, Uncertainty Avoidance), demographics (proportion of population above 65 years), and development (GDP per capita). Unlike in other studies, Collectivism was not significantly associated with ageism, controlling for covariates. Table 1 presents the results.

**Table 1.** Regressions of Ageism With Cultural Dimensions, Demographics, and Economics as Predictors and Covariates

	(1)	(2)	(3)	(4)
Log (GDP per capita)	−0.021	0.113	−0.015	−0.004
	(0.065)	(0.070)	(0.044)	(0.066)
Population above 65	1.230			−0.289
	(1.381)			(1.262)
Change in population above 65		−8.234		
(speed of aging)		(6.151)		
Power distance			0.014	0.010
			(0.199)	(0.209)
Collectivism			−0.240	−0.261
			(0.159)	(0.190)
Masculinity			0.865***	0.851***
			(0.196)	(0.214)
Uncertainty avoidance			0.387	0.417
			(0.346)	(0.385)
Long-term orientation			0.628***	0.651**
			(0.189)	(0.221)
*N*	20	20	18	18
*R* ^2^	0.088	0.136	0.792	0.793
Adjusted *R*^2^	−0.020	0.035	0.678	0.648

*Notes*: Standard errors in parentheses. Constant not shown.

*p < .1. **p < .05. ***p < .01.

A visual inspection of the residuals-versus-fitted plot for Model 4 did not suggest any heteroskedasticity. Similarly, White’s test (*p* > χ ^2^ = 0.389) and the Breusch–Pagan / Cook–Weisberg test (*p* > χ ^2^ = 0.326), also for Model 4, did not indicate any heteroskedasticity.

## Discussion

This is one of the first known studies to report associations between Masculinity, Long-term Orientation, and ageism across 20 countries. Higher levels of Masculinity and Long-term Orientation are associated with ageism, controlling for other cultural dimensions (Power Distance, Collectivism, Uncertainty Avoidance), demographics (percentage of elders above 65 years), and GDP per capita.

Highly masculine societies that emphasize competition and favor the strong and successful may systematically frame elders as weak, leading to the development of ageism at the societal level. This societal-level finding corroborates with qualitative research at the individual level that alluded to this link among older IT professionals (Comeau & Kemp, 2007) and older men with chronic conditions (Separavich & Oliveira, 2020). Our finding is consistent with studies that draw a link between ageism and ableism—defined as “a network of beliefs, processes and practices that produce a particular kind of self and body (the corporeal standard) that is projected as the perfect, species-typical and therefore essential and fully human” (Campbell, 2001, p. 44; Chivers, 2018; Overall, 2006; Sutter et al., 2017). The glorification of the capable and the degradation of the incapable are two sides of the same coin, therefore it is plausible that masculinity is linked to ageism.

On the other hand, societies with higher Long-term Orientation tend to be highly rational in an economic sense and may prefer to invest in the young with greater potential, instead of older persons who would be net consumers in the future. Our findings extend theoretical work on the societal trajectory of Long-term Orientation. While Long-term Orientation may be linked to innovation (Aurigemma & Mattson, 2019) and better cyber hygiene (Bukowski & Rudnicki, 2019), the (unintended) downside could be the systematic diminishment of groups with low future potential, such as older adults.

The lack of a statistically significant link between collectivism and ageism is inconsistent with the conclusion from North and Fiske’s (2015) meta-analysis. Our finding could be explained by Zhang and colleagues’ (2016) distinction between individualism at the personal and societal/cultural levels. Juxtaposing the World Values Survey data with priming experiments, they found that individual-level individualism was linked to ageism, but cultural-level individualism was not. Our findings are aligned as Hofstede’s (2001) measure of Collectivism is conceptualized at the societal/cultural level.

The lack of statistical significance for our control variable, GDP per capita, appear to support the inclusion of older workers in today’s labor markets as compared to the earlier industrialization period (Tavernier et al., 2019). Nevertheless, there is strong evidence to suggest that the substance of exclusion has not changed, though the form may have: ageism in the workplace is manifested consistently in hiring, promotion, and other human resource (HR) practices.

Of broader significance, our findings blunt the deterministic nature of ageism at the societal level. While countries with a faster aging population and higher percentage of older adults (Löckenhoff et al., 2009; North & Fiske, 2015) are more ageist, that is only one side of the ageism coin. Our study provides evidence that the cultural side is equally important—as shown in numerous studies from cognition (Dweck et al., 1995) to beauty perceptions (Broer et al., 2015; Heidekrueger, Sinno et al., 2017; Heidekrueger, Szpalski et al., 2017). The train has left the station on the demographic reality: Little can be done about slowing the pace of population aging or increasing the fertility rate that remains stubbornly low in many developed countries. Instead, cultural mindsets are more malleable, as priming studies have shown, and individual-level interventions to decrease interpersonal ageism have achieved considerable success (Lytle et al., 2020; Turner et al., 2018). Additionally, priming with implicit age stereotypes has been shown to effectively increase the physical performance of older adults (Levy et al., 2014). These individual-level efficacy trials need to be scaled up at the societal level, and our study provides the cultural considerations to do this effectively.

Drilling down to societal interventions that decrease ageism, future studies could focus on factors that mediate the culture–ageism relationship. In addition, societal-level interventions could be amplified by prioritizing countries high in Long-term Orientation and Masculinity. We hypothesize that countries high in Long-term Orientation and Masculinity are associated with more ageist health policies. Against this background, interventions could target policymakers in these societies to increase their awareness of ageism in health policies and public communications especially during the Covid-19 pandemic where elders are incessantly described as vulnerable. These research ideas will extend important studies showing institutional ageism in health policies formulated by governments and international organizations (Lloyd-Sherlock et al., 2016). Future studies should test the mediation hypothesis (e.g., Ng, Allore, et al., 2020) and design interventions targeted at policymakers to create a multiplier effect for their respective countries.

While this study circumvents the limitations of most survey studies that “provoke” responses rather than studying “naturally” occurring behavior (Hofstede, 2001, p. 4)—our study is not without limitations. The corpus compiled English sources, and did not include advanced aging countries like Japan. This is a significant limitation that will be addressed in future studies when we expand the corpus to other languages.

In conclusion, ageism has increased over 200 years (Ng et al., 2015) with a staggering 1-year price tag of $63 billion on the U.S. health care system (Levy et al., 2020). At this critical juncture, we need societal-level studies to distill the underpinnings of ageism. Across 20 countries, we show that cultural factors—Masculinity and Long-term Orientation—are linked to ageism, after adjusting for a country’s economic development and demographic trajectory. This important study lays the groundwork to design societal interventions to tackle our generation’s most insidious threats.
