# Corrigendum to: Ageism Linked to Culture, not Demographics: Evidence From an 8-Billion-Word Corpus Across 20 Countries

**DOI:** 10.1093/geronb/gbab146

**Published:** 2021-08-17

**Authors:** Reuben Ng, Jeremy W Lim-Soh

**Affiliations:** 1 Lee Kuan Yew School of Public Policy, National University of Singapore, Singapore; 2 Lloyds Register Foundation Institute for the Public Understanding of Risk, National University of Singapore, Singapore

In the article “Ageism Linked to Culture, not Demographics: Evidence From an 8-Billion-Word Corpus Across 20 Countries” [The Journals of Gerontology: Series B, gbaa181, https://doi.org/10.1093/geronb/gbaa181], errors were noted in co-author Jeremy W. Lim-Soh’s author listing. Jeremy W. Lim-Soh’s full name with degree should read: “Jeremy W. Lim-Soh, MA” instead of “Weizhong J Lim, MIS”. The ORCID ID was inadvertently omitted and has been instated.

